# Stimulation of homologous recombination in plants expressing heterologous recombinases

**DOI:** 10.1186/s12870-020-02545-7

**Published:** 2020-07-16

**Authors:** Abdellah Barakate, Ewan Keir, Helena Oakey, Claire Halpin

**Affiliations:** 1 Division of Plant Sciences, School of Life Sciences, University of Dundee at the JHI, Invergowrie, Dundee, DD2 5DA Scotland; 2 Current address: Cell and Molecular Sciences, The James Hutton Institute, Invergowrie, Dundee, DD2 5DA UK

**Keywords:** Intrachromosomal homologous recombination, Gene targeting, Gene editing, 2A, Translational-recoding, Pollen

## Abstract

**Background:**

Current excitement about the opportunities for gene editing in plants have been prompted by advances in CRISPR/Cas and TALEN technologies. CRISPR/Cas is widely used to knock-out or modify genes by inducing targeted double-strand breaks (DSBs) which are repaired predominantly by error-prone non-homologous end-joining or microhomology-mediated end joining resulting in mutations that may alter or abolish gene function. Although such mutations are random, they occur at sufficient frequency to allow useful mutations to be routinely identified by screening. By contrast, gene knock-ins to replace entire genes with alternative alleles or copies with specific characterised modifications, is not yet routinely possible. Gene replacement (or gene targeting) by homology directed repair occurs at extremely low frequency in higher plants making screening for useful events unfeasible. Homology directed repair might be increased by inhibiting non-homologous end-joining and/or stimulating homologous recombination (HR). Here we pave the way to increasing gene replacement efficiency by evaluating the effect of expression of multiple heterologous recombinases on intrachromosomal homologous recombination (ICR) in *Nicotiana tabacum* plants.

**Results:**

We expressed several bacterial and human recombinases in different combinations in a tobacco transgenic line containing a highly sensitive β-glucuronidase (GUS)-based ICR substrate. Coordinated simultaneous expression of multiple recombinases was achieved using the viral 2A translational recoding system. We found that most recombinases increased ICR dramatically in pollen, where HR will be facilitated by the programmed DSBs that occur during meiosis. DMC1 expression produced the greatest stimulation of ICR in primary transformants, with one plant showing a 1000-fold increase in ICR frequency. Evaluation of ICR in homozygous T2 plant lines revealed increases in ICR of between 2-fold and 380-fold depending on recombinase(s) expressed. By comparison, ICR was only moderately increased in vegetative tissues and constitutive expression of heterologous recombinases also reduced plant fertility.

**Conclusion:**

Expression of heterologous recombinases can greatly increase the frequency of HR in plant reproductive tissues. Combining such recombinase expression with the use of CRISPR/Cas9 to induce DSBs could be a route to radically improving gene replacement efficiency in plants.

## Background

The genome of all living organisms is continuously exposed to exogenous genotoxic agents and endogenous factors that can result in critical DNA lesions such as DNA double-strand breaks (DSBs). To maintain genome integrity, eukaryotic cells have evolved powerful and complex DNA damage repair mechanisms [1–3]. DSBs can be repaired by two main competing and partially overlapping pathways, homologous recombination (HR) and non-homologous end-joining (NHEJ) [4]. NHEJ is an error-prone repair pathway that can insert and/or delete short DNA sequences at the DSB site and result in frameshift and nonsense mutations, a feature widely exploited in recently developed ZFN (Zinc Finger Nuclease), TALEN (Transcription Activator-Like Effector Nuclease) and CRISPR (Clusters of Regularly Interspaced Short Palindromic Repeats) based gene editing technologies [5]. HR on the other hand is a conservative mechanism that results in reciprocal exchange of genetic information between two homologous DNA sequences or, more often, in gene conversion where the transfer is unidirectional. The prevalence of these two repair mechanisms depends on the species, cell type and even stage of cell cycle, with NHEJ being dominant in the majority of somatic cells while HR is most efficient in yeast, germline and mammalian embryonic stem cells [6].

Understanding DSB repair mechanisms could be extremely valuable for biotechnological approaches to crop improvement as well as to the treatment of cancer and gene therapy by improving gene replacement efficiency [7, 8]. Genetic modifications in plants have, until recently, relied exclusively on the random integration of transgenes by the host NHEJ pathway [9]. The perceived unpredictability of the process and the potential for the introduction of herbicide or antibiotic resistance markers within the final product make the genetically modified organism (GMO) less acceptable to the public. Using HR to make precise and targeted gene modifications (gene targeting), could be instrumental in alleviating some of these public concerns and in expanding the range of modifications that could be achieved. In plants, gene targeting (GT) has been elusive for a long time because NHEJ is the main route of DSB repair in plant cells, and the frequency of repair by HR tends to be orders of magnitude lower. Significant progress in targeting DSB to specific sites within genomes has been made within the past few years using synthetic endonucleases ZFN, TALEN and CRISPR-Cas [10–12]. Error-prone repair by NHEJ generates mutations, making it possible to knockout genes at will. Although the presence of DSBs is a prerequisite to HR stimulation [13], using CRISPR-Cas9 to facilitate integration of DNA at a target genomic locus by HR (i.e. GT to add, replace or modify a gene) is still extremely difficult. Spontaneous HR, in the range of 10^− 6^, is extremely low in somatic tissues that are frequently used in plant transformations and further stimulation of the HR machinery will be needed to make HR-mediated GT routinely achievable. Early work on HR in plants suggests that such stimulation might be achieved by expression of heterologous recombinases (i.e. genes involved in DNA repair and recombination) in plants. When expressed in *Nicotiana tabacum* plants, *E. coli* RecA and RuvC recombinases have been shown to increase intrachromosomal HR by 10- to 11-fold [14, 15] but RecA expression did not improve GT efficiency [16]. However, expression of the yeast RAD54 chromatin-remodelling gene was shown to increase the frequency of HR-mediated GT in Arabidopsis [17, 18]. Similarly, in mammals, overexpression of the eukaryotic RecA homolog, Rad51, induces HR by 20-fold [19] while expression of human BRCA2 in yeast increased HR by 2 to 2.5-fold [20].

In this study, we extend previous work by expressing six different heterologous recombinases, both individually and in combinations of two or three, in *Nicotiana tabacum*, and evaluate their influence on intrachromosomal HR. We used an artificial self-dissociating polyprotein to co-ordinately overexpress multiple recombinases from a single open reading frame [21]. A short peptide (20 aa) taken from the 2A region of foot-and-mouth disease virus separates distinct coding sequences within the polyprotein. This peptide effects, at its carboxy-terminus, efficient co-translational dissociation or ‘cleavage’ of the polyprotein into discrete protein products in plants [21, 22], an example of translational ‘recoding’. To facilitate evaluation of their effects on HR, recombinase constructs were introduced into transgenic tobacco plants containing a β-glucuronidase (uidA)-based intrachromosomal recombination (ICR) substrate (N1DC4 tobacco line) [23]. This transgenic line contains two truncated and partially overlapping uidA genes that flank, in direct orientation, a functional hygromycin resistance gene (hyg). ICR between these two defective uidA genes restores the functional marker that can be easily detected by histochemical staining for GUS activity. Here we report the frequency of ICR in seedlings and pollen of transgenic plants expressing single and multiple recombinases and validate the approach as a route towards potentially improving GT frequency in plants.

## Results

### Production of recombinase constructs

**Fig. 1:**
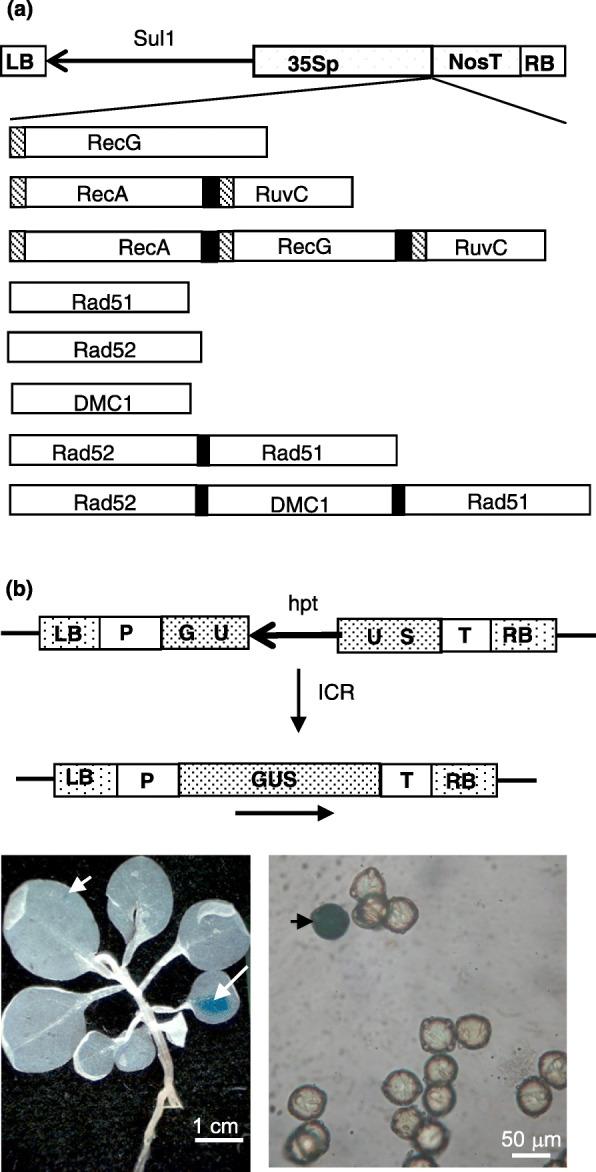
Recombinases constructs and intrachromosomal recombination (ICR) assay. **a** The coding sequences of bacterial (RecA, RecG and RuvC) and human (Rad51, Rad52 and DMC1) recombinases (white boxes) were amplified by PCR. Bacterial recombinases were tagged at their N-terminus with SV40 nuclear localisation signal (hatched box). The multigene constructs were made by inserting the 2A sequence from foot and mouth disease virus (black box) between different recombinases in a single open reading frame. These fragments (single and multiple genes) were inserted between the CaMV 35S promoter (35Sp) and nopaline synthase terminator (NosT) of pGSC plasmid containing left (LB) and right (RB) T-DNA borders and the sulphonamide resistant gene (Sul1) for plants selection. Different elements of these constructs schematics are not drawn to scale. **b** The transgene used as ICR substrate in the tobacco transgenic line N1DC4 is formed of two defective overlapping fragments of β-glucuronidase (GUS) separated by hygromycin resistance gene (hpt). ICR restore a functional GUS gene that can be detected by histochemical staining as blue spots on seedlings (left) and blue pollen (right)

We produced eight constructs in a pGEM®-T Easy vector to enable expression of single and multiple prokaryotic and eukaryotic recombinases to be initially checked by in vitro transcription and translation (Fig. 1a). RecA and RuvC that have been shown to increase ICR frequency when individually expressed in plants [14–16], were combined in one polyprotein construct. The prokaryotic RecG, that acts at a different step in DSB repair to RecA and RuvC to resolve Holliday junctions [24, 25], was used alone, and as a polyprotein with RecA and RuvC (RecA:RecG:RuvC). A nuclear localisation signal (NLS) was introduced at the N-terminus of all three bacterial recombinases. Similarly, constructs were prepared to express the eukaryotic orthologues of RecA (Rad51 and its meiotic homologue DMC1), and Rad52 (essential to DSB repair in yeast and mammals) individually or in polyproteins Rad52:Rad51 and Rad52:DMC1:RAD51. All constructs were later transferred to a plant transformation plasmid (see Methods) and expressed in tobacco plants (*Nicotiana tabacum*) from the constitutive CaMV 35S promoter. The host tobacco line used for transformation was the well-characterised transgenic line N1DC4 no. 29 [23, 26] that contains a single β-glucuronidase (GUS) based transgene as a substrate for ICR (Fig. 1b and Methods). Induced and spontaneous ICR events that restore a functional GUS gene can easily be monitored by histochemical staining of seedlings and pollen, counting blue spots (Fig. 1b) to determine the frequency of ICR.

### Testing of 2A polyprotein constructs in vitro

**Fig. 2:**
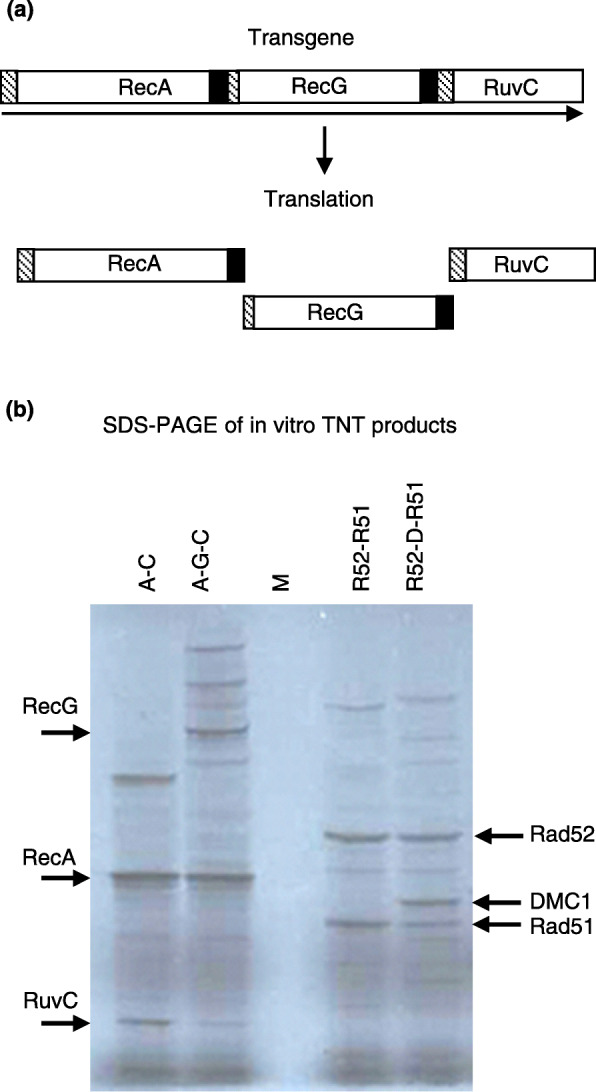
Test of plasmid constructs using in vitro transcription and translation. **a** An example of a construct containing multiple coding sequences (RecA, RecG and RuvC) in a single open reading frame (arrow) cloned in pGEM®-T Easy vector (Promega) is shown. During translation, the 2A sequence of foot and mouth disease virus (black box) allows the production of the individual proteins including their nuclear localisation signal (hatched box). **b** TNT® wheat germ lysate was performed with plasmid DNA of pRecA-2A-RuvC (A-C), pRecA-2A-RecG-2A-RuvC (A-G-C), pRad52-2A-Rad51 (R52-R51) and pRad52-2A-DMC1-2A-Rad51 (R52-D-R51). Arrows indicate individual products

Before introducing the constructs into plants, the polyprotein chimeric genes were transcribed and translated from the pGEM®-T Easy plasmids in which they had been initially assembled to check them for cotranslational cleavage. Although our previous work has shown that cleavage is more efficient when constructs are introduced into plants than it is in vitro [21], checking expression products in vitro is useful when antibodies are not available for detection of products *in planta*. SDS–PAGE of [^35^S]-methionine labelled products from a wheat germ TNT® system showed all the expected individual protein products were present, along with some high molecular weight uncleaved polyprotein. For example, translation of the RecA:RecG:RuvC polyprotein construct (Fig. 2a) results in discrete bands for RecA, RecG and RuvC, along with higher molecular weight bands for the RecA:RecG:RuvC polyprotein and partially ‘cleaved’ products RecA:RecG and RecG:RuvC (Fig. 2b). The signal intensity of RuvC and Rad51 products was still clear but less prominent when they were in the third position of their corresponding polyprotein. Overall the result in wheat germ indicated that the transgenes were suitable for expression in plants without the need for codon optimisation.

### Production and description of primary tobacco transformants

**Fig. 3:**
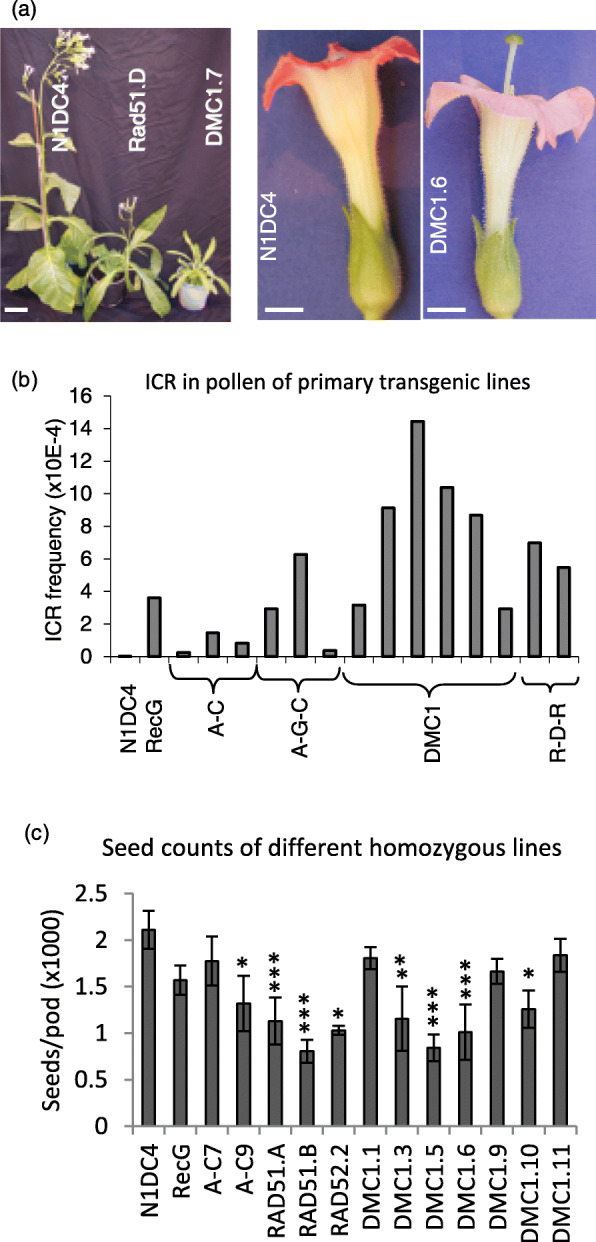
Growth and fertility of transgenic lines expressing various recombinases. **a)** Growth in the glasshouse of homozygous lines expressing recombinases and the control N1DC4 (left, scale bar = 10 cm) and flowers of some lines showing longer pistil (right, scale bar = 5 mm). **b** Intrachromosomal recombination (ICR) frequency in pollen. Pollen of 3 flowers of the control N1DC4 and T0 transgenic lines expressing recombinases was stained and scored for GUS activity. The value of ICR frequency in N1DC4 control was 0.013 × 10^− 4^. **c** The fertility in different homozygous lines compared to the control N1DC4. Data correspond to an average of 10 pods and error bars indicate standard errors. The transgenes are A-C, RecA-2A-RuvC; A-G-C, RecA-2A-RecG-2A-RuvC and R-D-R, Rad52-2A-DMC1-2A-Rad51

The constructs were introduced into tobacco N1DC4 and plants were regenerated under sulphonamide selection. Some transformants were severely affected in their development and were stunted with narrow leaves and changes in flower morphology (Fig. 3a), likely revealing pleiotropic effects of constitutive recombinase expression. Given some commonality of phenotype in different plants, this may reflect biochemical perturbation of developmental pathways as a consequence of recombinase expression, rather than random genomic mutation. Some lines produced very little pollen or presented pistils that were larger than the stamens, reducing their fertility. GUS staining of pollen from the first plants to regenerate showed that many had increased ICR frequency compared to control N1DC4 plants (Fig. 3b). This ICR snapshot demonstrated that all recombinases for which primary transformants were becoming available at this early stage (i.e. transformations were staggered and no transformants for Rad51, Rad52 and Rad51-Rad52 were yet available) strongly stimulated HR frequency to different degrees (Fig. 3b). Compared to the control N1DC4 plants with ICR frequency of 0.013 × 10^− 4^, DMC1 has the highest impact on ICR with frequencies ranging from 3.167 × 10^− 4^ to 14.442 × 10^− 4^ in six independent primary transformants, i.e. up to a 1000-fold increase.

### ICR frequency in seedlings and pollen of homozygote lines

Primary transformants continued to be collected for all constructs until no more were being produced i.e. not all primary transformants are represented in Fig. 3. Homozygote plant lines were produced from all primary transformants that had a single transgenic locus in order to increase the dosage of the transgene, and so that replicate plants per line could be analysed. This was necessary in order to take inter-plant variation into account and so that data could be statistically analysed. Unfortunately, some of the plants with highest ICR rates in Fig. 3b proved to have multiple insertions and could not easily be studied further. Fertility was scored for individual plants to assess the impact of different recombinases on meiosis since reduced fertility is a common phenotype when meiotic processes such as HR are manipulated. Compared to the N1DC4 control, most of the transgenic lines showed significant reductions in the number of seeds per pod, albeit to varying degrees (Fig. 3c). These changes in fertility provide some circumstantial evidence for continued expression of the heterologous recombinases in the homozygous plants, and possibly for different levels of expression in different plant lines. Unfortunately, the number of independent homozygous lines generated for each construct does not allow a robust statistical analysis.

**Fig. 4:**
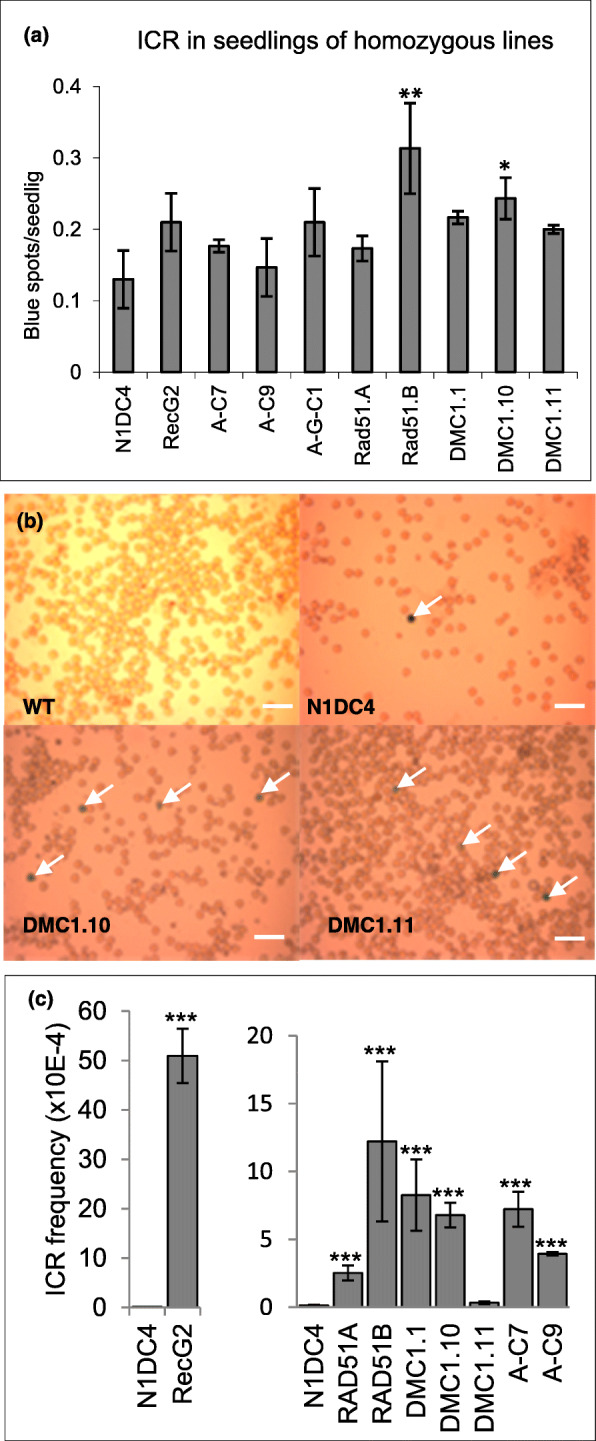
Intrachromosomal recombination (ICR) frequency in homozygous lines. **a** Six-week-old seedlings of the control N1DC4 and homozygous lines expressing recombinases were stained for GUS activity and the number of blue spots per seedling was scored. **b** Pollen of the controls wild type (WT) and N1DC4 and two homozygous lines expressing DMC1 in N1DC4 background were stained for GUS activity to detect blue pollen (arrow, scale bar = 100 μm). **c** ICR events (blue pollen) were scored for the control N1DC4 and different homozygous lines expressing recombinases. The average value of ICR frequency in N1DC4 control was (0.134 ± 0.04)× 10^− 4^. A-C, RecA-2A-RuvC construct. Data correspond to an average of 3–12 plants (3 flowers/plant) and error bars indicate standard errors

T2 seeds were germinated in three replicates on MS medium and grown for 6 weeks before the seedlings were stained for GUS activity in order to evaluate ICR in somatic vegetative tissue. Blue spots were scored and showed a moderate but significant increase of ICR for a couple of lines expressing the recombinases Rad51.B and DMC1 (Fig. 4a). Three to twelve homozygous seedlings of different transgenic lines were grown to maturity and their pollen stained for GUS activity (Fig. 4b) to evaluate ICR in meiotic reproductive tissue. Scoring of blue pollen grains revealed a much greater increase of ICR in pollen compared to seedlings of the same lines. Of the limited number of homozygous lines that could be produced, the one homozygote RecG expressing line had the strongest stimulation of ICR by 380-fold. RecA:RuvC expressing plants showed an increase of 29- to 54-fold. Except for the DMC1.11 line which only showed a moderate increase (2-fold), the human recombinases Rad51 and DMC1 stimulated ICR in pollen by a factor of 18.8- to 91-fold.

## Discussion

Gene editing and gene targeting (GT) are powerful tools that will be very useful in precise gene manipulation in gene therapy and crop improvement. The development of CRISPR/Cas9 endonuclease systems has made genome editing routinely feasible in higher eukaryotes including plants [10]. CRISPR has been used with variable efficiency to target both endogenous [12, 27, 28] and artificial reporter genes [29] in plants using transient and stable transformation methods [30]. However, these manipulations are so far mainly limited to gene mutagenesis by NHEJ repair of targeted DSB, while gene replacement by HR is still not efficient for routine usage. CRISPR has been used recently to integrate the selectable marker nptII into the ADH1 gene of Arabidopsis by HR but the GT frequency remains low [28, 31]. Gene replacement was improved by adopting dual-sgRNA/Cas9 [32] and sequential transformation [33] strategies in Arabidopsis or simultaneous targeting of adjacent introns in rice [34]. Gene replacement was also used to generate glyphosate resistant cassava plants [35] and ARGOS8 variants in maize [36]. Most reported experiments have been performed in Arabidopsis and rice where transformation is particularly efficient and may not reflect the bulk of plant species. In addition, reproductive meiotic tissue is the target of Arabidopsis floral dip transformation whereas transformation methods for most species target non-meiotic tissues. In the current work we pave the way towards an efficient gene replacement tool for all transformable plants by successfully increasing HR via expression of multiple bacterial and human proteins that are required at different steps of DSB repair.

Rad52 is essential in the choice of DSB repair pathway by HR in yeast and animals [37, 38] but the plant Rad52 ortholog has only been found more recently [39]. Prokaryotic RecA and its eukaryotic orthologs Rad51 and DMC1 bind single strand DNA of DSB ends and initiate homology searching, promoting repair by HR. Although expression of human Rad51 and Rad52 reduced HR in mammalian cells [40], yeast Rad52 increased the efficiency of GT 37-fold in human cells while reducing NHEJ [38]. These conflicting findings could be related to varying levels of protein expression and the nature of the recombination substrates used in these studies. Here, we expressed human Rad51, its meiotic counterpart DMC1, and Rad52 individually or in combination in tobacco. Both Rad51 and DMC1 significantly stimulated ICR in pollen from homozygous transgenic plants by a factor of 19-fold and 91-fold respectively, with moderate increases in ICR also seen in seedlings. Due to the presence of multiple insertions and problems with fertility in the recombinase-expressing transgenics, plants expressing all three recombinases (Rad52:DMC1:Rad51 or R-D-R Fig. 3) could not be followed beyond the primary transformant generation but, although this construct also significantly increased ICR frequency, there was no evidence for it being better than expression of DMC1 alone. One primary transformant plant expressing DMC1 displayed an increased ICR frequency in pollen of 1000-fold. However, homozygous progeny from this plant did not produce sufficient pollen to enable replicated statistically validated data to be generated.

The translocase RecG is crucial in DNA repair of stalled replication forks [41, 42] and DSBs [43, 44], promoting branch migration of Holliday junctions [24, 25, 45]. Eukaryotes do not seem to have a nuclear RecG homologue [46, 47]; instead, yeast and human Rad54 have been shown to promote branch migration of synthetic Holliday junctions (HJs) in vitro [48]. The expression of yeast Rad54 in Arabidopsis increased GT by up to 10-fold [17, 18]. Here we show that heterologous expression of the bacterial RecG can increase ICR frequency by 380-fold, which, if replicated in a full GT assay, could have significant implications for facilitating gene replacement in plants.

The final recombinase in our constructs, *E. coli* RuvC, is necessary for the resolution of Holliday junctions [49]. RecA and RuvC have been previously expressed in tobacco plants showing an increase of ICR of 10- to 11-fold [14, 15]. Here we expressed them together and showed increases in ICR of 29-fold and 60-fold in two independent homozygous lines, suggesting at least additive and possibly synergistic effects of expressing both recombinases.

Our results showing that ICR is much more stimulated in pollen compared to seedlings reflects the likelihood that recombination events happened during meiosis (although extra events during microsporogenesis could not be excluded). It is possible that HR could therefore be increased even further by using meiotic promoters that provide stronger expression in meiotic tissue than the CaMV 35S promoter used here. For example, the barley DMC1 promoter has recently been shown to be inflorescence-specific and useful in transgenic experiments targeting meiotic tissues [50]. Expression of heterologous recombinases from meiotic promoters might have the added benefit of eliminating the stunting and aberrant phenotypes we saw in vegetative tissue in some lines due to constitutive expression. Sterility might not be avoided using a meiotic promoter but might be reduced using an inducible promoter. A tuneable tetracycline inducible promoter, for example, could be used to express the recombinases at optimal levels and avoid their cytotoxicity. Alternatively, in a system aimed at transiently increasing HR to promote CRISPR-mediated GT, the recombinase-expressing transgene cassette could be rapidly removed by outcrossing once the desired GT had occurred. Outcrossing is greatly simplified using our 2A-polyprotin system as all recombinases and also other elements of a gene replacement system (e.g. the CRISPR-Cas9 or other targeted nuclease) can be encoded in a single transgene for facile removal as a unit.

ICR might be most stimulated in meiotic tissue because such tissues are already primed for DSB repair by HR. DNA double-strand break induction has been shown to stimulate recombination by up to 2000-fold in yeast [51]. More recently, CRISPR was used to stimulate ICR frequency in Arabidopsis [52]. It will be interesting to challenge our transgenic lines expressing multiple recombinases with DSB-inducing CRISPR nuclease to see whether further HR stimulation can be achieved, which potentially could improve gene replacement efficiency. Yeast Rad52 expression in chicken DF-1 cells increased gene replacement by 3-fold in two CRISPR/Cas9 targets [53]. In rice, gene replacement is now achievable using a strong negative selection marker and high-throughput transformation and screening [54]. However, this system is so far unique to rice and not applicable to all plants. Our ICR/2A system in pollen will help to quickly validate the effect of multiple recombinase expression on HR and build the network of regulators of GT. Further improvements could be achieved by inhibiting the competing NHEJ pathway and combining all of these improvements in a CRISPR-guided system for targeting double stranded breaks.

## Conclusions

Opportunities for CRISPR/Cas deployment in plant biotechnology are currently limited to gene editing applications but would be greatly expanded by the addition of full gene replacement (gene targeting) technology. Here we show that expression of several bacterial or eukaryotic recombinases or combinations of recombinases can dramatically increase ICR in tobacco. Greatest increases were seen with the single recombinases DMC1, RecG and Rad51. If these stimulations of HR translate to full gene targeting assays where the efficiency of CRISPR/Cas is also deployed to generate targeted double strand breaks, it could pave the way for a revolutionary gene replacement methodology for higher plants.

## Methods

### Bacterial and human recombinases cloning

The coding sequences of bacterial recombinases RecA, RecG and RuvC were amplified by polymerase chain reaction (PCR) using *E. coli* chromosomal DNA as template and the corresponding primers (Table 1) containing convenient restriction sites. The PCR product were cloned in pGEM®-T Easy vector (Promega) and sequenced. SV40 large T-antigen nuclear localisation signal (NLS) [14] was made with two complementary oligonucleotides (Table 1) and inserted at the 5′ end of the cloned bacterial recombinases. The coding sequences of human recombinases Rad51, Rad52 and DMC1 were PCR amplified using as template pFB530 [55], pFB581 [37] and phDMC1 [56], respectively and the corresponding primers (Table 1). Polyprotein constructs were assembled in pGEM®-T Easy transcription vector as described previously [22].

**Table 1:** List of oligonucleotides

Oligonucleotide^**a**^	Restriction sites^**b**^	Sequence (5′ - 3′)^**c**^
CaMVp-F	KpnI	GGGTACCCAAAGATTCAAATAGAGGACCT
rbcSL-R	HindIII; NdeI; NcoI	CCAAGCTTCCATATGAGCCATGGAAGCCATTTTTCTCAC
CaMVT-F	EcoRI	GGAATTCGTCCGCAAATCACCAGTCTCTC
CaMVT-R	SphI; SacI	CGCATGCGAGCTCGGTCACTGGATTTTGGTTTTAGG
NLS-F1	BamHI; SmaI	GATCCATGATGGGGACTCCTCCTAAGAAGAAGCGTAAGGTTCCC
NLS-R1	SmaI; BamHI	GGGAACCTTACGCTTCTTCTTAGGAGGAGTCCCCATCATG
NLS-F2	ApaI; SmaI	CATGATGGGGACTCCTCCTAAGAAGAAGCGTAAGGTTCCC
NLS-R2	ApaI; SmaI	GGGAACCTTACGCTTCTTCTTAGGAGGAGTCCCCATCATGGGCC
RecA-F	SmaI	TCCCCCGGGATGGCTATCGACGAAAACAAACAG
RecA-R	XbaI	GCTCTAGAAAAATCTTCGTTAGTTTCTGCTAC
RecG-F1	SmaI	TCCCCCGGGATGAAAGGTCGCCTGTTAGATGCTG
RecG-R1	XbaI	GCTCTAGACGCATTCGAGTAACGTTCCGTCTC
RecG-F2	HindIII	CCCAAGCTTGGGCCCATGATGGGGACTCC
RecG-R2	EcoRI	CGGAATTCTTACGCATTCGAGTAACGTTC
RuvC-F	SmaI	TCCCCCGGGATGGCTATTATTCTCGGCATTGATC
RuvC-R	PstI	GGCTGCAGTTAACGCAGTCGCCCTCTCGCCAGGTTCAG
Rad51-F	ApaI	GGGGCCCATGGCAATGCAGATGCAGCTTG
Rad51-R	PstI; SmaI	AACTGCAGCCCGGGTCAGTCTTTGGCATCTCCCAC
Rad52-F	SalI	GCGGTCGACATGTCTGGGACTGAGGAAGC
Rad52-R	SphI	GTAGCATGCGTAAGATGGATCATATTTCC
DMC1-F	ApaI	AAGGGCCCATGAAGGAGGATCAAGTTGTGGCG
DMC1-R	PstI; SphI	AACTGCAGGCATGCCTCCTTGGCATCCCCAATTCCTCC

^**a**^ F and R at the end of the oligonucleotide’s name indicate forward and reverse orientations, respectively. ^**b**^ The ends of annealed forward and reverse NLS primers are compatible for cloning into the indicated restriction sites. ^**c**^ The restriction sites are underlined in the sequence

### In vitro expression

Polyprotein constructs were used with wheat germ transcription–translation system (TNT®, Promega) according to the manufacturer’s instructions in the presence of [^35^S]-Methionine (Amersham, Buckinghamshire, UK). Radiolabelled protein products were separated in 10% SDS–PAGE [57] and detected by autoradiography.

### Plant expression vector pGSC

The EcoRV fragment containing sulphonamide resistant gene under nopaline synthase transcriptional signals (nos-Sul) was inserted into HpaI close to the left border of pGreenII 0000 [58] to make pGS plasmid. pS/ntRecA was digested with EcoRI and HindIII and the fragment containing CaMV 35S promoter, the 5′ leader of the ribulose-bisphosphate carboxylase/oxygenase (Rubisco) small subunit (rbcS), SV40 nuclear localisation and the N-terminus of RecA was transferred into pSP73 (Promega), yielding pSP73-35S:N-RecA. This plasmid was digested with KpnI, the ends were rendered blunt and religated to obtain pSP73-35S:N-RecAΔKpnI. This plasmid was used as template to amplify CaMV 35S promoter and the 5′ leader of rbcS using PCR and oligonucleotides containing KpnI (5′) and HindIII (3′) restriction sites (Table 1). The PCR fragment was digested with KpnI and HindIII and cloned into pLBR19 to replace the double CaMV 35S promoter [59]. The large CaMV terminator (735 bp) in pLBR19 was also replaced with a smaller version (221 bp) using PCR amplification and oligonucleotides containing EcoRI (5′) and SacI-SphI (3′) (Table 1), yielding p35S. This plasmid was digested with KpnI and SacI and the released CaMV 35S cassette was cloned into pGS to make pGSC binary vector. All bacterial and human recombinases constructs in pGEM®-T Easy were transferred into pGSC plasmid using convenient restriction enzymes.

### Plant transformation

The constructs in pGSC binary vector were transferred into *Agrobacterium tumefaciens* strain LBA4404 containing pSoup plasmid by electroporation method. Agrobacterium clones were used to transform tobacco seedlings of N1DC4 line as described by Abbott et al. [60]. Tobacco N1DC4 transgenic line was a gift by Professor Barbara Hohn, Friedrich Miescher Institute for Biomedical Research, Switzerland and Professor Holger Puchta, Karlsruhe Institute of Technology, Germany. The transgenic lines, selected in the presence of 100 mg/l of sulphonamide, were transferred to the glasshouse after two rounds of rooting. At maturity pollen and seeds of the obtained primary transformants were collected.

### Transgene segregation and seed scoring.

T1 seeds were plated on MS medium in the presence of 100 mg/l of sulphonamide and scored for resistance after 2 weeks growth under 16 H light regime. The lines showing 3:1 segregation ratio were selected to make homozygotes. Six resistant seedlings per line were grown in the glasshouse and T2 seeds collected. These seeds were screened in the presence of sulphonamide to detect homozygous lines (100% resistance). Three plants per homozygous lines and N1DC4 control were grown in the glasshouse and their pollen and seeds were collected. The seeds of ten dry pods per plant were pooled and their number estimated by weight method.

### Intrachromosomal recombination (ICR) assay

N1DC4 is a homozygous line containing β-glucuronidase (GUS) based transgene as a substrate for Intrachromosomal recombination (ICR) [23, 25]. The transgene is formed of two defective overlapping GUS fragments in direct orientation and separated by hygromycin resistance gene (hpt). ICR restores a functional GUS gene that can be detected by histochemical staining as blue spots on seedlings and blue pollen. To determine the number of ICR events in somatic cells, six-week-old seedlings were stained for GUS activity [23] and the number of blue spots recorded under a binocular microscope. To monitor ICR in pollen, dehiscent anthers of three flowers were combined in 1.5 ml microfuge tube containing 1 ml of GUS staining buffer supplemented with 20% methanol to inhibit endogenous GUS activity. The pollen concentration was determined using haemocytometer and the recombination rate was calculated based on the number of blue pollens.

### Statistical analysis

A separate linear regression model was fitted for each of the responses (blue spots per seedling, number of seeds per pod and ICR in pollen) with the genotypes included in the model as an explanatory factor. Using corner point parameterisation of the genotype factor, each of the test genotypes was compared to the control genotype N1DC4 and was taken to be significantly different from the control if the t statistic had an associated *P*-value less than 0.05.

## Data Availability

All transgene constructs used in this work and generated raw data of ICR counts are available and can be obtained from Professor Claire Halpin.

## Abbreviations

ICR: Intrachromosomal recombination
NLS: Nuclear localisation signal;
GT: Gene targeting
CRISPR: Clusters of regularly interspaced short palindromic repeats
HR: Homologous recombination
DSB: Double-strand break
GUS: β-Glucuronidase
NHEJ: Non-homologous end joining
